# Self-Reduction in Proximal Humerus Fractures through Upright Patient Positioning: Is It up to Gravity?

**DOI:** 10.3390/diagnostics12092096

**Published:** 2022-08-29

**Authors:** Sam Razaeian, Christian Krettek, Nael Hawi

**Affiliations:** 1 Trauma Department, Hannover Medical School, Carl-Neuberg-Str. 1, 30625 Hannover, Germany; 2 Hannover Humerus Register (HHR), Traumastiftung gGmbH, Carl-Neuberg-Str. 1, 30625 Hannover, Germany; 3 Orthopädisch-Chirurgische Praxisklinik Braunschweig (OCP), Mauernstraße 35, 38100 Braunschweig, Germany

**Keywords:** proximal humerus fracture, conservative treatment, nonoperative, self-reduction, gravity

## Abstract

Background: The self-reduction in proximal humerus fractures (PHFs) remains a poorly explored myth, and it was rarely investigated in the past. One of the oldest hypotheses suggests that gravity and the weight of the affected arm alone are driving forces, which facilitate a self-reducing potential in PHFs. However, thus far, clear radiographic evidence for this theory is missing in the literature. This study aimed to investigate the immediate effect of upright patient positioning on self-reducing of PHFs. Methods: Between November 2019 and November 2020, seven consecutively bedridden but mentally competent patients were admitted to our emergency department with an acute proximal humerus fracture. Within routinely attempts of closed reductions under the control of an image converter (C-arm), immobile patients were mobilized into an upright sitting position on a stretcher while the affected arm was immobilized in a sling. Fluoroscopic controls were performed before and after upright positioning. Changes in the head-shaft angle (HSA), as well as the medial hinge index (MHI), were determined on plain true anteroposterior (AP) fluoroscopic images. Results: In all cases, upright patient positioning had an immediate self-reducing effect. This effect could be seen in five out of seven cases for both HSA and MHI. Changes in HSA and MHI averaged 21.2° and 0.25, respectively. Mean deviation from an ideal, anatomic HSA of 135° decreased through upright positioning from 25.5° to 13.8°. Mean deviation from an ideal, anatomic MHI of 1 decreased through upright positioning from 0.28 to 0.19. Conclusions: Upright patient positioning might contribute immediately to the self-reduction in PHF through the force of gravity. This underlines the importance of being aware of patients’ position when interpreting X-ray images within treatment decision-making processes.

## 1. Introduction

Proximal humeral fractures (PHFs) are a common injury, representing approximately 6% of all adult fractures. The incidence of PHFs has been increasing over the past few decades, with an aging population as well as the associated increase in osteoporosis and low-energy falls from standing height [1,2,3,4,5]. 

It is well known that the majority of PHFs can be treated nonoperatively [3,6,7,8,9,10,11,12]. Generously dimensioned amounts of fragment displacement and angulation reported to be compatible with good functional results [13,14,15], suggesting a self-reducing potential of PHFs, which remained a poorly explored myth and was mentioned only occasionally in the literature [14,16,17,18,19]. The scattered observed phenomenon (for example, a displaced four-part fracture at diagnosis according to the most commonly used Neer classification may turn spontaneously into a minimally displaced one-part PHF at a one-week follow-up visit) has generated several hypotheses [17]. One of the oldest studies suggests that gravity is the driving force, which aids in bringing the affected arm to the side into a favorable orthograde position, pulls the shaft downwards, and counteracts displacing forces as that of the pectoralis major traction to medial, facilitating the medial hinge’s reduction [17,18,20]. 

DePalma and others already hypothesized decades ago that the weight of the affected arm alone with approximately 10–15 pounds would be sufficient for reducing or correcting the fracture completely in many cases and would additionally not accentuate malalignments of the fracture [18,21]. Codman’s pendulum exercises in a so-called hanging loose position within the nonoperative follow-up treatment are founded on this theory [17,22]. 

This is why Einarsson already emphasized half a century ago the importance of patient mobilization out of the bed as far as possible due to missing reducing traction forces by gravity during bedridden conditions [18]. 

However, thus far, clear radiographic evidence for this theory is missing in the literature. 

## 2. Materials and Methods

### 2.1. Patients

This study prospectively analyzed gathered data of an ongoing observational registry studies (Hannover Humerus Registry—HHR). The protocol is registered at ClinicalTrials.gov (NCT03060876).

**Figure 1:**
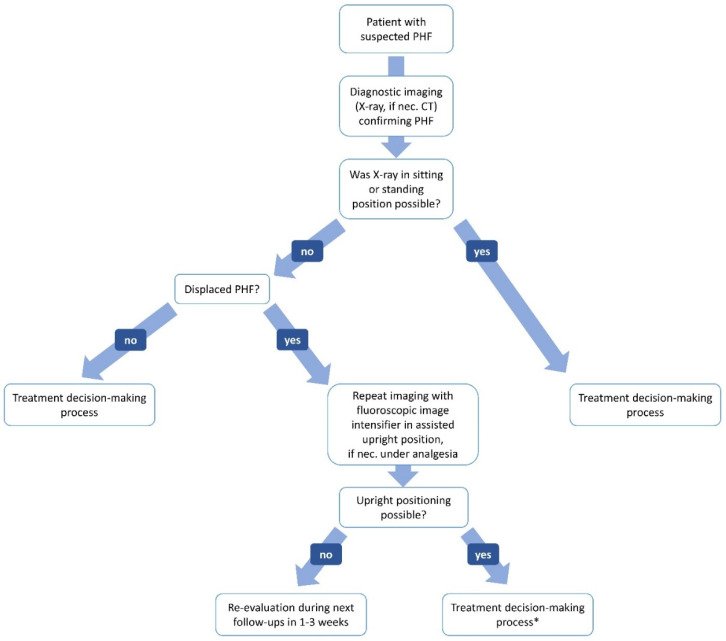
Patients’ journey in the form of an algorithm. * Further self-reducing potential should be taken into account.

Between November 2019 and November 2020, seven consecutively bedridden but mentally competent patients were admitted to our emergency department with an acute proximal humerus fracture. Within routine attempts of closed reduction under the control of an image converter (C-arm) (Ziehm Solo, Serialno. 52554, Ziehm Imaging GmbH, Nürnberg, Germany), the immobile patients were mobilized into an upright sitting position on a stretcher while the affected arm was immobilized in a sling. Fluoroscopic controls were performed before and after upright positioning and prior to further reduction attempts as well as the application of any additional medical reduction-aids. Figure 1 shows the patient’s journey in the form of an algorithm during the above-mentioned study period.

Fluoroscopic records as well as variables such as age, gender, and reason for being bedridden were retrospectively analyzed. 

All patients were part of an observational registry study (Hannover Humerus Registry—HHR) (no. 3222-2016), which was carried out in accordance with the Ethical standards of the 1964 Declaration of Helsinki as updated in 2004, and provided written informed consent.

### 2.2. Fluoroscopic Analyses

**Figure 2:**
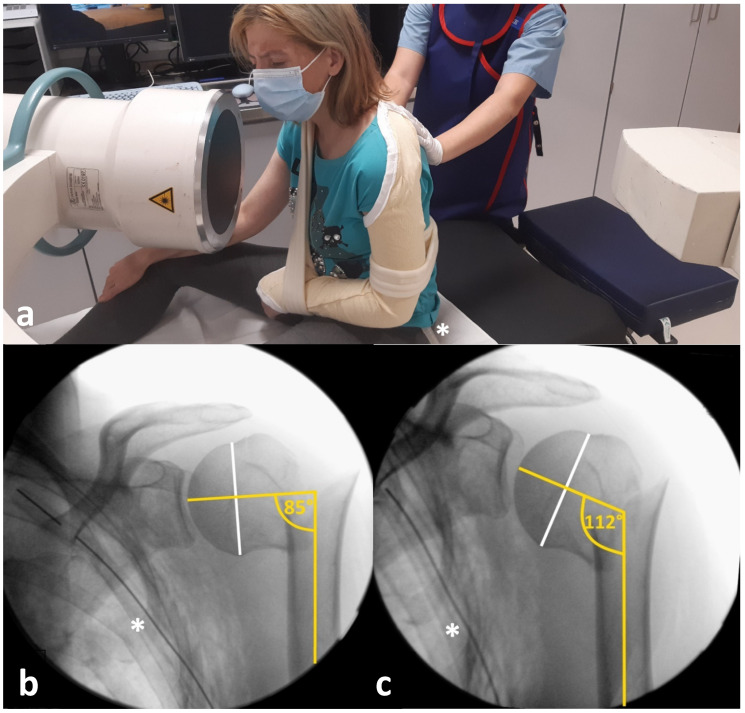
Patient no. 5 is mobilized into an upright sitting posture by a nurse assistant while the affected left shoulder is immobilized in a sling The ipsilateral chest tube is marked with an asterisk (*) (**a**). Fluoroscopic images of patient no. 5 in lying (**b**) and in an upright sitting posture (**c**), showing an immediate self-reducing effect with respect to HSA through patient mobilization. A white line is drawn from the superior to the inferior border of the articular surface. A perpendicular yellow line is drawn to this line and through the center of the humeral head. The angle between this line and another yellow line bisecting the humeral shaft is measured as HSA. An ipsilateral chest tube due to serial rib fractures with pneumothorax is marked with an asterisk (*).

Changes in the head-shaft angle (HSA), as well as the medial hinge (MH), before and after mobilizing the patient to an upright position were determined on plain true anteroposterior (AP) fluoroscopic images with Visage 7.1 (Visage Imaging Inc.—San Diego, CA, USA). During fluoroscopic imaging, the palm of the hand was facing and touching the belly while the affected arm was immobilized in a sling. HSA was measured as described by Agudelo et al. [23]. A line was drawn from the superior to the inferior border of the articular surface. Afterwards, a perpendicular line was drawn to this line and through the center of the humeral head. The angle between this line and the line bisecting the humeral shaft was measured as HSA (Figure 2b,c). Anatomic HSA was defined as 135° [24].

**Figure 3:**
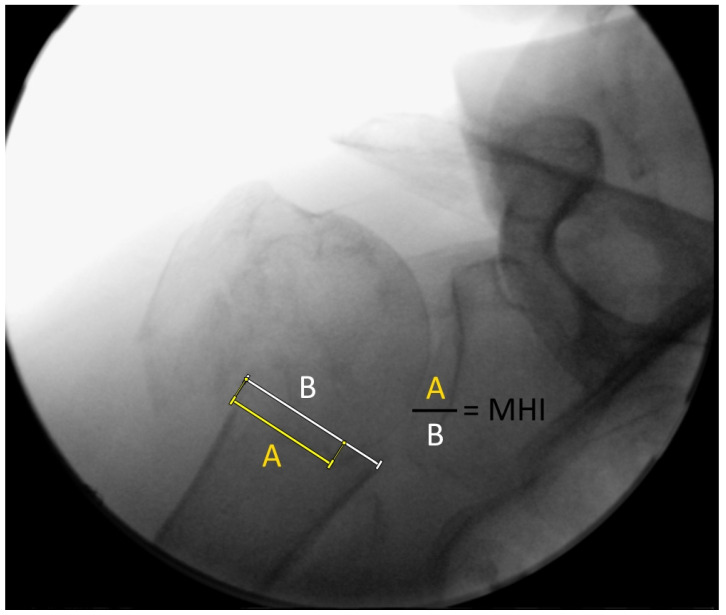
Fluoroscopic measurement of the medial hinge index (MHI). ‘A’ is defined as a line from the most proximal and lateral point of the humeral shaft to the most medial humeral head articular surface. ‘B’ is defined as a line from the most proximal and lateral point to the most proximal and medial point of the humeral shaft.

For measurements of the medial hinge, a so-called medial hinge index (MHI) was calculated in relation to the humeral shaft diameter to compare unreferenced C-arm images (Figure 3).

### 2.3. Exemplary Case Presentation

**Figure 4:**
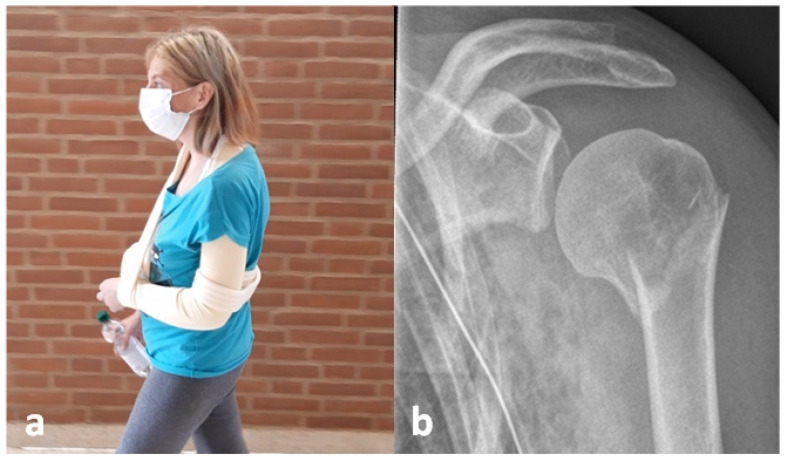
The patient was, several days later, able to walk independently (**a**). An anteroposterior X-ray control before chest tube removal in an upright standing position indicates a nearly anatomic HSA (**b**).

Figure 2 exemplarily shows a 40-year-old female study participant who sustained a varus displaced 2-part fracture involving the surgical neck according to the Neer classification system after a fall from a height of about 2 m from the balcony [13]. The HSA was initially altered by 50° from an ideal anatomic HSA of 135°. However, the X-ray images were taken in lying position due to immobilizing pain caused by concomitant ipsilateral serial rib fractures with pneumothorax. Fluoroscopic images show an immediate self-reducing effect of assisted upright positioning on the HSA (Figure 2b,c). Several days later, the patient was able to walk independently. An anteroposterior X-ray control before chest tube removal in an upright standing position indicates a further self-reducing potential and a nearly anatomic HSA (Figure 4b).

Values less than 1 were defined as a displacement of the medial hinge. Values greater than or equal to 1 were considered as a reduced and favorable fracture configuration. 

All fractures were classified according to the AO Foundation/Orthopaedic Trauma Association (AO/OTA) fracture classification system [25]. The classification system was abbreviated to the following categories: A (extraarticular unifocal), B (extraarticular bifocal), and C (articular).

### 2.4. Statistical Analyses

Descriptive statistics, including means, standard deviations, and ranges were calculated for assessing changes in HSA and MHI. Data analysis was performed with SPSS 26.0 (IBM, Armonk, New York, NY, USA).

## 3. Results

Seven patients were analyzed. The average age was 69 years (range, 40–83 years). Table 1 provides details about gender, reasons for immobility, and fracture type of each patient.

In all cases, upright patient positioning had an immediate self-reducing effect. This effect could be seen in five out of seven cases for both HSA and MHI. 

Table 2 shows changes of HSA, MHI, and the residual amount of malalignment of each patient using fluoroscopic assessments in lying (highlighted red) and upright sitting positions (highlighted green). Changes in HSA and MHI averaged 21.2° and 0.25, respectively. The mean deviation from an ideal, anatomic HSA decreased through upright positioning from 25.5° to 13.8°. The mean deviation from an ideal, anatomic MHI of 1 decreased through upright positioning from 0.28 to 0.19. 

## 4. Discussion

This is the first study to investigate the immediate effect of upright patient positioning on the self-reduction in proximal humerus fractures. The fact that this phenomenon could be observed in all cases without any need of active reduction or pendulum exercises confirms one of the oldest hypotheses that the arm alone and traction force by gravity would be sufficient to reduce PHFs and can additionally not accentuate malalignment [18,21]. 

In addition, it underlines the importance of patient mobilization out of the bed as far as possible as a fundamental element of nonoperative fracture treatment [18]. 

The actual verification of this old hypothesis makes it meaningful not only to label X-ray images accordingly but also to be aware of the patients’ position while the X-ray image is taken within therapeutic decision-making processes (Figure 1), as it is common when interpreting X-rays of the chest or spine (Figure 2) [24,26,27,28,29,30]. 

In addition, this point should be taken into consideration when assessing additional computer tomographic images usually made in lying position. 

Currently, there are only a scarce amount of studies dealing with this unexplained phenomenon, which is indicated often enough in the past [16]. 

While Lucas-Championnière reported, more than a century ago, that all non-impacted fractures involving the neck of the humerus might reduce themselves by allowing the arm hang down with the forearm supported in a sling, Einarsson extended this suggestion to greatly displaced and impacted fractures based on casuistries [18]. Similarly to DePalma and Cautilli, Wallace stated his observation of many patients with displaced PHFs in which the fracture’s configuration improved after simply suspending the arm in a collar and cuff sling [19]. 

Keene confirmed this assumption in the case of two-part fractures with more than 55° or 1.5 cm of displacement. These fractures would be able to reduce themselves spontaneously when immobilized in a sling or cuff and collar. In his view, this effect would be attributed to the long head of the bicep’s tendon acting as an internal sling controlling both fragments. This is why, he concluded, that deformities beyond proposed limits, as described for example by Neer, may initially be accepted when treating these fractures [14]. However, all these assumptions were only experience-based. Recently, Fang et al. reported about self-reductions and is, thus far, the only existing clinicoradiological analysis on this specific issue. They retrospectively described seven patients with a three-part valgus impacted fracture patterns that underwent spontaneously self-reduction with an average decrease in HSA from 169° to 141° without any active reductions. The authors were unable to definitively explain how and, in particular, at what exact time this unique observation could occur due to the retrospective design of their study and inconsistent follow-up intervals between radiographs, but they postulated that transient deltoid weaknesses could be factors in facilitating self-reductions [16]. 

Aguado et al. retrospectively assessed fracture displacement in PHFs managed with conservative treatments after early mobilization and a home-based self-exercise program [17]. The authors observed changes in HSA from varus into valgus (55° ± 23° to 42° ± 22°) within the normal range of anatomic values and a reduction in the medial hinge. While X-ray images on the day of diagnoses showed medial displacements of the diaphysis (+1 ± 6 mm), the diaphysis healed laterally to the head (−0.6 ± 6 mm; *p* = 0.005), meaning an impaction of the shaft in the humeral head in a more stable configuration at the final follow-up visit [17]. Contrarily to these findings, we could observe such a self-reducing potential not during a longer follow-up period but as an immediate result of upright patient positioning in acute fracture settings.

This study has some limitations. As a pure case series with only seven patients, a transfer of these findings to all fracture patterns remains unfeasible. Moreover, only changes in coronal alignment have been analyzed, while a sagittal view is missing in order to reduce unnecessary radiation exposure. In addition, it is questionable to what extent the amount of self-reduction would change decision making with respect to treatment regimens. 

Furthermore, the observed fluoroscopic findings have been investigated only at one moment, while further follow-up measurements are missing and are under observation as part of an ongoing prospective study. In the form of an only momentary snapshot, the actual self-reducing potential remains unknown, and it is not answered with this study; this should be investigated in future studies.

## 5. Conclusions

Upright patient positioning might contribute immediately to the self-reduction in PHF through the force of gravity. This underlines the importance of being aware of patients’ position when interpreting X-ray images within treatment decision-making processes.

## Figures and Tables

**Table 1:** Details about age, gender, reasons for immobility, and fracture type of each patient.

Patientno.	Age	Gender	AO/OTA	Days after Injury	Reason for Immobility
1	59	m	B	1	Immobilizing pain
2	67	f	B	5	Multimorbidity and erysipelas of both legs
3	83	f	A	0	Immobilizing pain, sarcopenia
4	79	f	A	1	Immobilizing pain, sarcopenia
5	40	f	A	1	Immobilizing pain and serial rip fractures with pneumothorax
6	76	m	A	0	Immobilizing pain due to multiple bruises of the lower extremities
7	79	m	C	7	Obesity, multimorbidity, and immobilizing pain due to multiple bone bruises of the lower extremities

**Table 2:** Measured values of each patient using fluoroscopic assessments. LP = lying position (highlighted red); UP = upright sitting position (highlighted green); HSA = head-shaft angle; MHI = medial hinge index; ∆ HSA = difference of HSA between LP and UP; ∆ MHI = change of MHI between LP and UP; *n*.*m*. = not measured. ^1^ Anatomic deviation is defined as the residual amount of malalignment considering an HSA of 135 ° and MHI of 1 as ideal. ^2^ Self-reducing is defined as any change towards ideal values.

Patient-no.	HSA in		∆ HSA	Anatomic Deviation ^1^ in		MHI in		∆ MHI	Anatomic Deviation ^1^ in		Self-Reducing ^2^?	
	LP	UP		LP	UP	LP	UP		LP	UP	HSA	MHI
1	154.1°	137.7°	16.4°	19.1°	2.7°	0.86	1.03	0.17	0.14	0.03	Yes	Yes
2	157.9°	148°	9.9°	22.9°	13°	1.27	1	0.27	0.27	0	Yes	Yes
3	142°	125.2°	16.8°	7°	9.8°	0.51	0.74	0.23	0.49	0.26	No	Yes
4	146°	131.3°	14.7°	11°	3.7°	0.67	0.82	0.15	0.33	0.18	Yes	Yes
5	85°	112°	27°	50°	23°	n.m.	n.m.	n.m.	n.m.	n.m.	Yes	n.m.
6	91.5°	110.5°	19°	43.5°	24.5°	1.33	1.22	0.11	0.33	0.22	Yes	Yes
7	160°	115.1°	44.9°	25°	19.9°	0.87	1.45	0.58	0.13	0.45	Yes	Yes
**Mean**			**21.24°**	**25.5°**	**13.8°**	**0.92**	**1.04**	**0.25**	**0.28**	**0.19**		

## Data Availability

Not applicable.

## References

[1] Palvanen M., Kannus P., Niemi S., Parkkari J. (2006). Update in the epidemiology of proximal humeral fractures. Clin. Orthop. Relat. Res..

[2] Court-Brown C.M., Caesar B. (2006). Epidemiology of adult fractures: A review. Injury.

[3] Jawa A., Burnikel D. (2016). Treatment of Proximal Humeral Fractures: A Critical Analysis Review. JBJS Rev..

[4] Launonen A.P., Lepola V., Saranko A., Flinkkilä T., Laitinen M., Mattila V.M. (2015). Epidemiology of proximal humerus fractures. Arch. Osteoporos..

[5] Kristiansen B., Barfod G., Bredesen J., Erin-Madsen J., Grum B., Horsnaes M.W., Aalberg J.R. (1987). Epidemiology of proximal humeral fractures. Acta Orthop. Scand..

[6] Burkhart K.J., Dietz S.O., Bastian L., Thelen U., Hoffmann R., Müller L.P. (2013). The treatment of proximal humeral fracture in adults. Dtsch. Arztebl. Int..

[7] Court-Brown C.M., Cattermole H., McQueen M.M. (2002). Impacted valgus fractures (B1.1) of the proximal humerus. The results of non-operative treatment. J. Bone Jt. Surg. Br..

[8] Court-Brown C.M., Garg A., McQueen M.M. (2001). The translated two-part fracture of the proximal humerus. Epidemiology and outcome in the older patient. J. Bone Jt. Surg. Br..

[9] Court-Brown C.M., McQueen M.M. (2004). The impacted varus (A2.2) proximal humeral fracture: Prediction of outcome and results of nonoperative treatment in 99 patients. Acta Orthop. Scand..

[10] Rangan A., Handoll H., Brealey S., Jefferson L., Keding A., Martin B.C., Goodchild L., Chuang L.H., Hewitt C., Torgerson D. (2015). Surgical vs nonsurgical treatment of adults with displaced fractures of the proximal humerus: The PROFHER randomized clinical trial. JAMA.

[11] Launonen A.P., Sumrein B.O., Reito A., Lepola V., Paloneva J., Jonsson K.B., Wolf O., Ström P., Berg H.E., Felländer-Tsai L. (2019). Operative versus non-operative treatment for 2-part proximal humerus fracture: A multicenter randomized controlled trial. PLoS Med..

[12] Murray I.R., Amin A.K., White T.O., Robinson C.M. (2011). Proximal humeral fractures: Current concepts in classification, treatment and outcomes. J. Bone Jt. Surg. Br..

[13] Neer C.S. (1970). Displaced proximal humeral fractures. I. Classification and evaluation. J. Bone Jt. Surg. Am..

[14] Keene J.S., Huizenga R.E., Engber W.D., Rogers S.C. (1983). Proximal humeral fractures: A correlation of residual deformity with long-term function. Orthopedics.

[15] Neer C.S. (1970). Displaced proximal humeral fractures. II. Treatment of three-part and four-part displacement. J. Bone Jt. Surg. Am..

[16] Fang C., Kwek E.B.K. (2017). Self-reducing proximal humerus fractures. J. Orthop. Surg..

[17] Aguado H.J., Ariño B., Moreno-Mateo F., Bustinza E.Y., Simón-Pérez C., Martínez-Zarzuela M., García-Virto V., Ventura P.S., Martín-Ferrero M. (2018). Does an early mobilization and immediate home-based self-therapy exercise program displace proximal humeral fractures in conservative treatment? Observational study. J. Shoulder Elb. Surg..

[18] Einarsson F. (1958). Fracture of the upper end of the humerus: Discussion based on the follow-up of 302 cases. Acta Orthop. Scand..

[19] Young T.B., Wallace W.A. (1985). Conservative treatment of fractures and fracture-dislocations of the upper end of the humerus. J. Bone Jt. Surg. Br..

[20] Duparc F. (2013). Malunion of the proximal humerus. Orthop. Traumatol. Surg. Res..

[21] Depalma A.F., Cautilli R.A. (1961). Fractures of the upper end of the humerus. Clin. Orthop..

[22] Codman E.A. (1990). Rupture of the supraspinatus tendon. 1911. Clin. Orthop. Relat. Res..

[23] Agudelo J., Schürmann M., Stahel P., Helwig P., Morgan S.J., Zechel W., Bahrs C., Parekh A., Ziran B., Williams A. (2007). Analysis of efficacy and failure in proximal humerus fractures treated with locking plates. J. Orthop. Trauma.

[24] Jeong J., Bryan J., Iannotti J.P. (2009). Effect of a variable prosthetic neck-shaft angle and the surgical technique on replication of normal humeral anatomy. J. Bone Jt. Surg. Am..

[25] Meinberg E.G., Agel J., Roberts C.S., Karam M.D., Kellam J.F. (2018). Fracture and Dislocation Classification Compendium-2018. J. Orthop. Trauma.

[26] McKiernan F., Faciszewski T. (2003). Intravertebral clefts in osteoporotic vertebral compression fractures. Arthritis Rheum..

[27] Puddy E., Hill C. (2007). Interpretation of the chest radiograph. Contin. Educ. Anaesth. Crit. Care Pain.

[28] Toyone T., Toyone T., Tanaka T., Wada Y., Kamikawa K., Ito M., Kimura K., Yamasita T., Matsushita S., Shiboi R. (2006). Changes in vertebral wedging rate between supine and standing position and its association with back pain: A prospective study in patients with osteoporotic vertebral compression fractures. Spine.

[29] Díez-Ulloa M.A., Gallego-Goyanes A. (2015). Prognostic value of an immediate lateral standing X-ray with a TLSO in patients with a thoracolumbar burst fracture. Rev. Esp Cir. Ortop. Traumatol..

[30] Mehta J.S., Reed M.R., McVie J.L., Sanderson P.L. (2004). Weight-bearing radiographs in thoracolumbar fractures: Do they influence management?. Spine.

